# Long term persistence of clonal malaria parasite *Plasmodium falciparum* lineages in the Colombian Pacific region

**DOI:** 10.1186/1471-2156-14-2

**Published:** 2013-01-07

**Authors:** Diego F Echeverry, Shalini Nair, Lyda Osorio, Sanjay Menon, Claribel Murillo, Tim JC Anderson

**Affiliations:** 1 Department of Entomology, Purdue University, 901 West State Street, West Lafayette, IN, 47907, USA; 2 International Center for Medical Research and Training, CIDEIM, Carrera 125 # 19-225 Av. La María, Cali, Colombia; 3 Department of Genetics, Texas Biomedical Research Institute, 7620 NW Loop 410, San Antonio, Texas, USA; 4 Grupo de Epidemiología y Salud Poblacional, GESP, School of Public Health, Faculty of Health, Universidad del Valle, Calle 4B # 36-00, Cali, Colombia; 5 Virginia Commonwealth University, Richmond, VA, 23220, USA

**Keywords:** *Plasmodium falciparum*, Colombia, Clonality, Relatedness, Persistence, Genotypic richness, Population structure, SNPs, Linkage disequilibrium, Association studies

## Abstract

**Background:**

Resistance to chloroquine and antifolate drugs has evolved independently in South America, suggesting that genotype - phenotype studies aimed at understanding the genetic basis of resistance to these and other drugs should be conducted in this continent. This research was conducted to better understand the population structure of Colombian *Plasmodium falciparum* in preparation for such studies.

**Results:**

A set of 384 SNPs were genotyped in blood spot DNA samples from 447 *P. falciparum* infected subjects collected over a ten year period from four provinces of the Colombian Pacific coast to evaluate clonality, population structure and linkage disequilibrium (LD). Most infections (81%) contained a single predominant clone. These clustered into 136 multilocus genotypes (MLGs), with 32% of MLGs recovered from multiple (2 – 28) independent subjects. We observed extremely low genotypic richness (R = 0.42) and long persistence of MLGs through time (median = 537 days, range = 1 – 2,997 days). There was a high probability (>5%) of sampling parasites from the same MLG in different subjects within 28 days, suggesting caution is needed when using genotyping methods to assess treatment success in clinical drug trials. Panmixia was rejected as four well differentiated subpopulations (*F*_*ST*_ = 0.084 - 0.279) were identified. These occurred sympatrically but varied in frequency within the four provinces. Linkage disequilibrium (LD) decayed more rapidly (r^2^ = 0.17 for markers <10 kb apart) than observed previously in South American samples.

**Conclusions:**

We conclude that Colombian populations have several advantages for association studies, because multiple clone infections are uncommon and LD decays over the scale of one or a few genes. However, the extensive population structure and low genotype richness will need to be accounted for when designing and analyzing association studies.

## Background

Studies of parasite genetic structure are of practical importance in low transmission malaria regions for a number of reasons. First, long term persistence of identical multilocus genotypes (MLGs) may generate an upward bias in PCR-corrected estimates of treatment failure rates and genetic data can be used to estimate the size of this bias. Second, the longer range of linkage disequilibrium (LD) expected in such populations can simplify genetic mapping since genomic regions influencing traits of interest may be detected with a lower density of markers. At the same time, however, population structure may introduce bias into such analyses. Therefore, detailed evaluations of both LD and population structure are needed to design appropriate association studies in such regions. Third, while low malaria transmission areas are responsible for a small proportion of the world’s malaria cases, such regions appear to play a disproportionate role in evolution of drug resistance. For example, resistance to chloroquine (CQ) has evolved twice in South America, with additional origins in Southeast Asia and Papua New Guinea [1]. However, there is no evidence that CQ resistance has arisen in Africa, where ~90% of the world malaria cases occur, and a similar story also holds for resistance to pyrimethamine [2]. Finally, effective control measures have reduced malaria transmission in many hyperendemic regions of sub-Saharan Africa. Over time we expect that malaria parasite population structure in these regions may become more similar to that currently observed in Southeast Asia and South America.

Malaria is endemic in 20 countries in Central and South America, with Brazil and Colombia accounting for ~65% of all reported cases [3]. Colombia has approximately 100,000 malaria cases per year and >60% of the Colombian population is at risk for malaria. Malaria transmission is unstable with all age groups affected, and nearly all malaria cases are symptomatic [4]. Using genotyping of the antigenic genes merozoite surface protein (*msp*) *1* and *2*, parasite populations from Quibdó city (Chocó, Colombia) were shown to be genetically depauperate with low levels of multiclonal infections [5]. In fact, a single haplotype accounted for more than 60% of all the 390 samples studied. A subset of 56 of these samples were further studied with five microsatellites confirming very limited variation: 13 different haplotypes were found among these 56 isolates [5]. This low genetic diversity is consistent with other molecular study performed in Panguí (Chocó) [6], Turbo and Zaragoza (Antioquia, Colombia) [7] and with studies performed in South America, but contrasts with similar studies in Africa [8-11].

Most work on South American *P. falciparum* has employed antigen or microsatellite markers. As data from these markers are difficult to compare between laborato-ries, we sought to use methods that are more portable. Here, we examined the population genetics and population structure of *P. falciparum* infections using 384 SNPs in *P. falciparum* sampled from the Pacific region of Colombia between 1993–2007. We used these data to: (1) test methods for genome wide SNP typing using limited parasite DNA from dried bloodspots; (2) determine the number of parasite populations present within the region sampled; (3) measure the persistence of identical multilocus genotypes in time and space, and (4) examine patterns of LD across the genome. Our central goals are to determine how parasite population structure impacts interpretation of clinical drug trials and the design of genetic association analyses in this region of Colombia.

## Methods

### Parasite samples

**Figure 1:**
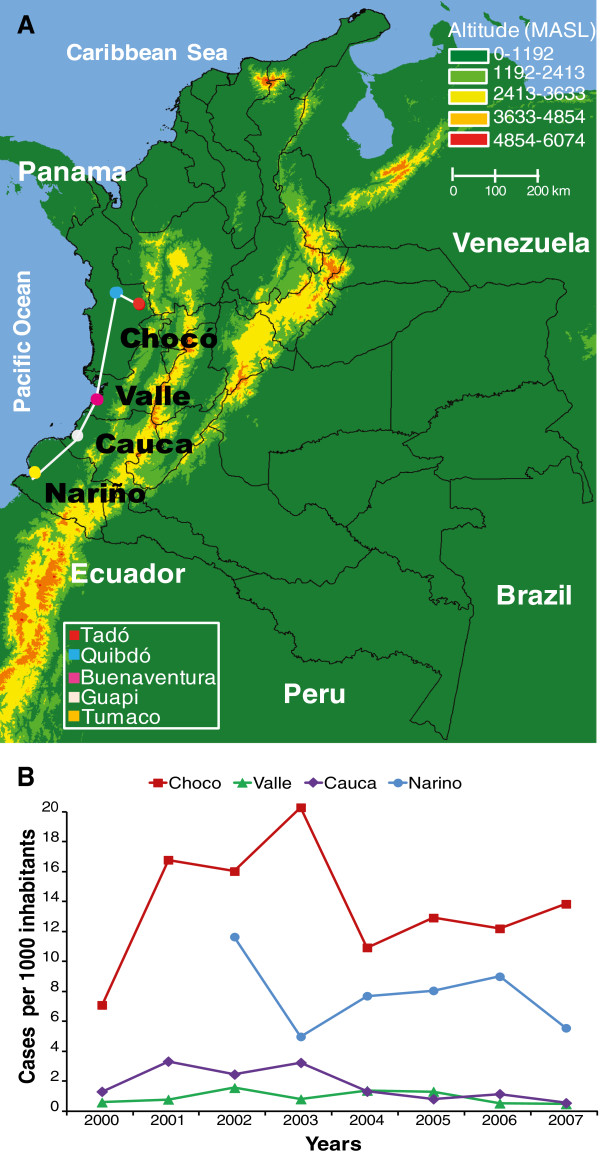
**Sampling of *Plasmodium falciparum *populations and malaria epidemiology in the Colombian Pacific region. A)** Map of Colombia - South America showing four malaria endemic provinces located in the Pacific region: Chocó, Valle, Cauca and Nariño, and the cities where samples were collected. About 90% of the population in this region located between the Pacific Ocean and the Western branch of the Andean Mountains are Afro-Colombians. Lines represent distance between sample collection sites: Quibdó-Tadó: ~66 km; Quibdó-Buenaventura: ~253 km; Buenaventura-Guapi: ~149 km and Guapi-Tumaco: ~109 km. **B)** Malaria endemicity in the four states defined by the annual parasite incidence (API) between 2000 – 2007. Data was not available from 2000 – 2001 in Nariño. For complementary information see Table 1.

Microscopically confirmed *P. falciparum* samples included in this study came from five cities of the Colombian Pacific region: Tadó and Quibdó in the province of Chocó, Buenaventura in Valle, Guapi in Cauca, and Tumaco in Nariño (Figure 1A, Table 1). Collectively, these four provinces account for up to 75% of *P. falciparum* cases reported in Colombia [4]. Strong differences in the transmission of falciparum malaria are observed among these locations (Figure 1B and Table 1). Finger-prick blood spot samples were collected on filter paper (Whatman 3 MM, Whatman International, Maidstone, England) from 447 Colombian *P. falciparum* samples. Samples were obtained from primary infections of subjects with uncomplicated malaria who took part in studies conducted by CIDEIM from 1993 to 2007. Ninety five percent were obtained from fresh blood and 5% from samples previously adapted to culture. Informed consent was obtained from all the subjects enrolled, as approved by CIDEIM Institutional Review Board (IRB). We included nine *P. falciparum* reference strains: 3D7 (collected in the Netherlands), 7G8 (Brazil), Dd2 (Indochina/Laos), FCB (Southeast Asia), FCC2 (China), HB3 (Honduras), K1 (Thailand), Santa Lucia (Guatemala) and V1/S (Vietnam) from the Malaria Research and Reference Reagent Resource Center - MR4 (http://www.mr4.org), as controls for the genotyping methodology.

**Table 1:** **Summary of *Plasmodium falciparum *samples, epidemiology data and malaria vectors in the Colombian Pacific region**

**State**City	**Location**	**Inhabitants (% per state)**^**a**^	**Multiclonal infection/primary cases**	**Number of monoclonal samples**	**Competent vectors in the state**^**b**^	**Malaria falciparum cases per state per year**^**c**^	**Number of monoclonal samples collected per state per year**^**d**^
**Chocó** Quibdó Tadó	5°41’19”N-76°39’33”W5°16’03”N-76°33’57”W	388476109121(28%)15962(4%)	25/102 **(25%)**	77		2000: 2616	1997: 2
					An. darlingi*	2001: 6383	2000: 6
					*An. nuneztovari*	2002: 6089	2001: 13
					An.pseudopuntipennis*	2003: 7745	2002: 1
					An. neivai *	2004: 3863	2004: 14
					An. puntimacula*	2005: 4441	2005: 6
						2006: 4320	2006: 13
						2007: 5025	2007: 22
						2000: 985	1993: 4
**Valle** Buenaventura	3°25’52”N-77°03’17” W	4161125 324207 (8%)	8/57 **(14%)**	49	*An.albimanus,*	2001: 1767	1994: 1
					*An. nuneztovari*	2002: 5003	1999: 4
					An.pseudopuntipennis*	2003: 1859	2000: 1
					An. neivai*	2004: 4369	2002: 1
					An. puntimacula*	2005: 4115	2004: 13
						2006: 745	2005: 15
						2007: 643	2006: 10
						2000: 1209	
**Cauca**Guapi	2°34’17”N-77°53’15”W	1268937 28649 (2%)	11/78 **(14%)**	67		2001: 3770	
					*An. albimanus*	2002: 2670	1999: 1
					*An. nuneztovari*	2003: 3657	2000: 1
					An. neivai*	2004: 1177	2003: 65
					An. puntimacula*	2005: 466	
						2006: 948	
						2007: 198	
						2000: ND	1999: 1
**Nariño** Tumaco	1°48’34”N-78°45’46”W	1541956 161490 (11%)	31/163 **(19%)**	132		2001: ND	2000: 2
					*An. albimanus*	2002: 17417	2001: 10
					An. neivai*	2003: 7085	2002: 12
					An. puntimacula*	2004: 6250	2003: 56
						2005: 9992	2004: 2
						2006: 9245	2005: 23
						2007: 7994	2006: 1
							2007: 25
**Total**			75/400 **(18.75%)**	325			

^a^Information according to the 2005 Colombian National Census [12]; ^b^Reports are based on field studies, underlined species are considered primary vectors in Colombia [13,14]; ^c^Information provided by SIVIGILA – Instituto Nacional de Salud (INS) of Colombia; ^d^Include only the 325 monoclonal samples analyzed; ND: Not Determined; *Complexes of species.

### DNA extraction and whole genome amplification (WGA)

For each sample, four or six mm punches of the blood spot were used for DNA recovery and purification. We used a three step process to prepare DNA for SNP genotyping: (1) DNA was recovered from blood spots using the Gensolve kit (GenVault Corporation, Carlsbad, CA); (2) the QIAamp DNA Blood Mini Kit (Qiagen, Valencia, CA) was used to purify recovered DNA and concentrated using a speed vacuum (20 min at 45°C) to achieve a final volume ~10 μL, and (3) the illustra GenomiPhi V2 DNA amplification kit (GE Healthcare, Piscataway, NJ) was used to amplify 1 μL of gDNA. The final volume of DNA was 60 μL (eluted in TE buffer) and DNA was quantified using a NanoDrop 1,000 spectrophotometer (Thermo Fisher Scientific Inc, Wilmington, DL).

### GoldenGate SNP genotyping

A custom GoldenGate® genotyping assay was designed using 384 SNPs obtained from coding genes. These included 126 synonymous and 258 non-synonymous SNPs (Additional file 1). The SNPs were selected using the query system on PlasmoDB (http://www.plasmodb.org) [15]. We selected SNPs that were polymorphic (with a minor allele observed in at least two Central and South American samples), situated >50 kb from telomeres to exclude antigenic genes, that were assigned a score of >0.6 using the Illumina Design Tool (ILLUMINA® ADT, Illumina, San Diego, CA). The SNPs were genotyped using the GoldenGate® genotyping assay [16], following Illumina protocols with 50 ng of starting DNA in 5 μL of water for each sample. Clustering was done using the BeadStudio package (Illumina, San Diego, CA). We defined clusters of parasites with alternative bases (wildtype or mutant) at each SNP: parasites falling in between these two extremes were assumed to be mixed infections.

### Clonality assessment, relatedness and persistence

We calculated the proportion of alleles shared (*ps*) between all pairwise comparisons of all single clone parasites following the procedure of Anderson et al. [17,18]. The genetic distance matrix was estimated using ARLEQUIN v 3.1 software [19], a UPGMA phenogram was constructed using the metric 1 - *ps* with PHYLIP v 3.69 [20], and plotted using FIGTREE software v 1.3.1 [21]. Parasite samples identical at all genotyped SNPs (i.e. clones) were identified by inspecting the terminal branches of the phenogram. Parasites with unique haplotypes at all tested SNPs are referred as multilocus genotypes (MLGs). We calculated genotypic richness (R), a measure of the proportion of unique genotypes in the population sample as R = ^*G - 1*^*/*_*n - 1*_, were *G* is the total number of MLGs found in *n* samples [22]. We computed the probability of sampling the same MLG from different infected patients separated by different time intervals within each province. This analysis was done using the clonal sub-range fix option included in the GenClone 2.0 software [23,24], with time intervals relevant for *in vivo* antimalarial efficacy studies.

### Population structure assessment

This analysis was conducted using one representative of each MLG after exclusion of multiple infections. We used the Bayesian model–based clustering algorithm implemented in STRUCTURE v 2.1 [25,26] to investigate genetic structure. We used an admixture model with correlated allele frequencies [26], with a Markov Chain Monte Carlo (MCMC) of 10,000 ‘burn-in’ steps followed by 100,000 interactions and 20 replicate runs at each *K* value (1 to 16). The optimal *K* partition was estimated following the STRUCTURE directions and the methodology proposed by Evanno et al. [27].

### Estimation of linkage disequilibrium

HAPLOVIEW software was used to compute linkage disequilibrium (LD) [28]. We calculated LD between each pairwise combination of linked markers and plotted the relationship between physical distances and LD. To measure the extent of LD, we used the correlation coefficient (r^2^). Analyses were completed for both in the complete set of unique haplotypes and for the subpopulations identified by STRUCTURE software. We estimated the level of LD between unlinked markers (SNPs in different chromosomes for each subpopulation) in order to estimate the background LD caused by small sample size in the populations, or relatedness, as was performed by Van Tyne et al. [29].

## Results

### Efficient SNP genotyping using Goldengate® assay from blood spots

Parasitemias of genotyped samples ranged from <0.01% (including two samples with 80 parasites/μL) to 1.09% of infected red blood cells. Following multiple displacement amplification of DNA extracted from blood spots [30], we obtained an average of ~12 μg of DNA per sample with an OD_260_:OD_280_ ~1.8 although most of the amplified DNA came from human origin. All 17 samples adapted to culture were successfully genotyped.

**Figure 2:**
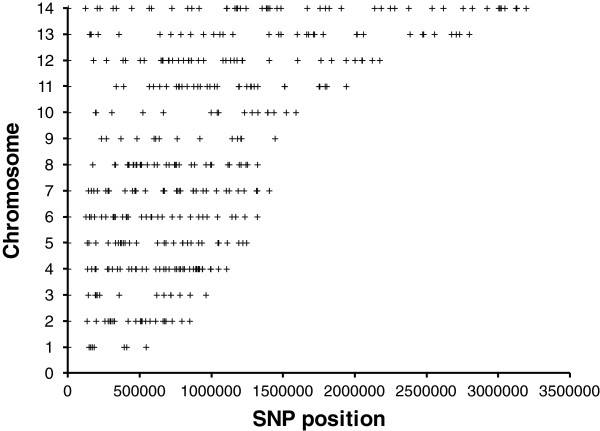
**Distribution of Single Nucleotide Polymorphisms (SNPs) used for genotyping.** Location of 384 SNPs through the 14 chromosomes of *P. falciparum*. There were 6, 22, 12, 39, 27, 30, 36, 34, 14, 15, 31, 37, 33, and 48 SNPs on chromosomes 1 – 14, respectively.

A total of 384 SNPs distributed across the 14 chromosomes of the parasite were genotyped (Figure 2, Additional file 1), with an average of one SNP per 60 kb. Forty three SNPs (11%) were rejected due to poor quality (poorly resolved or scored in <90% samples) and 34 (9%) were non variable SNPs (minor allele frequency - MAF <5%), leaving 307 (80%) informative SNPs with MAF >5% (Additional file 1). We genotyped 447 primary infections and nine reference strains. Thirty two samples (7%) with parasitemias between 150 and 18,200 parasites/μL had incomplete SNP data (>10% of missing data) and 15 samples were excluded due to conflict with identification. A total of 400/447 (89.5%) Colombian Pacific coast primary infections were included in the statistical analyses (Table 1).

The reference strains DD2, HB3, 7G8 and Santa Lucia were evaluated twice in different plates. The validation rate (concordance level) between the same reference strains in different plates to the total SNP calls was high, 99.1, 99.4, 99.7 and 96%, respectively (Additional file 2). We also compared concordance between GoldenGate SNP calls and those in the genome sequence data avai-lable at PlasmoDB. Several discrepancies were found. For FCB, DD2, 3D7, HB3 and 7G8, we observed high concordance (between 95.1 to 99.4%), while for K1, V1/S, FCC2 and Santa Lucia concordance was lower (88.1 to 92.6%) (Additional file 2). As the GoldenGate® genotyping is a robust technique, SNP discordances may be explained by low sequencing coverage (1.25X) of these samples [29].

### Clonality assessment reveals strong relatedness and long term persistence

**Figure 3:**
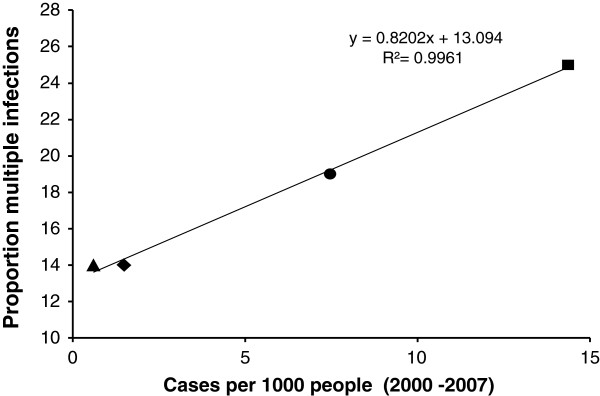
**Association between malaria transmission and multiple clone infection.** Correlation between number of multiclonal infections and malaria transmission intensity in the Colombian Pacific region. Square, circle, diamond and triangle represent isolates from Chocó, Nariño, Cauca and Valle respectively.

Polyclonal infections (>1 clone of *P. falciparum* per sample) were defined as the samples with >10 heterozygous SNP calls and were found in 75/400 (19%) samples (Table 1). For those samples, the mean number of heterozygous SNPs was 27.7 (range 11 – 66). We observed 25, 14, 14, and 19% multiple infections in Chocó, Valle, Cauca and Nariño provinces respectively. There was a strong positive correlation between carriage of multiple genotypes and transmission intensity (r^2^ = 0.99) (Figure 3).

**Figure 4:**
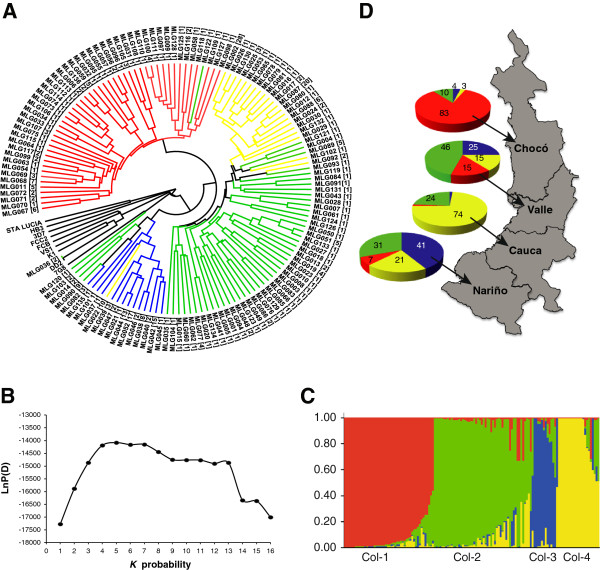
**Determination of multilocus genotypes and population structure from Colombian *P. falciparum *from the Pacific region. A**) UPGMA phenogram built with 325 monoclonal samples, using the distance metric 1 - proportion of shared alleles. The labels at the branch tips indicate Colombian parasites MLGs or laboratory controls from MR4. The number in parenthesis shows the number of identical parasites within a MLG (n = 136) and the colors of the branches indicate the four populations identified by the program STRUCTURE. **B)** Plot of Var [LnP(D)] vs. *K* probability showing the best *K* subpopulation partition at *K* = 4; for complementary information see Additional file 3. **C)** Output from STRUCTURE software. Each MLG is represented by a vertical line and each color represents a different subpopulation; many MLGs showed mixed ancestry (length of the colored lines indicate the estimated proportion of membership of each MLG to that subpopulation). **D)** Distribution of the four subpopulations across the provinces at the Colombian Pacific region. The frequency of each subpopulation is shown as a percentage.

A subset of 325 monoclonal samples from Chocó, Valle, Cauca and Nariño were retained for further analysis (Table 1). These samples had 250 informative SNPs, as several SNPs were not informative (MAF <5%) after exclusion of polyclonal infections. The 325 monoclonal parasites comprised 136 unique MLGs, with 44 (32%) represented by MLGs infecting more than one patient (range 2 – 28) (Figure 4A). MLG 036 comprises two culture-adapted samples from Quibdó and Tadó that were indistinguishable from the Dd2 reference strain from Southeast Asia. Contamination during *in vitro* adaptation (W2, the Dd2 progenitor, is cultured at CIDEIM since 2000) or DNA manipulation may explain this observation.

**Figure 5:**
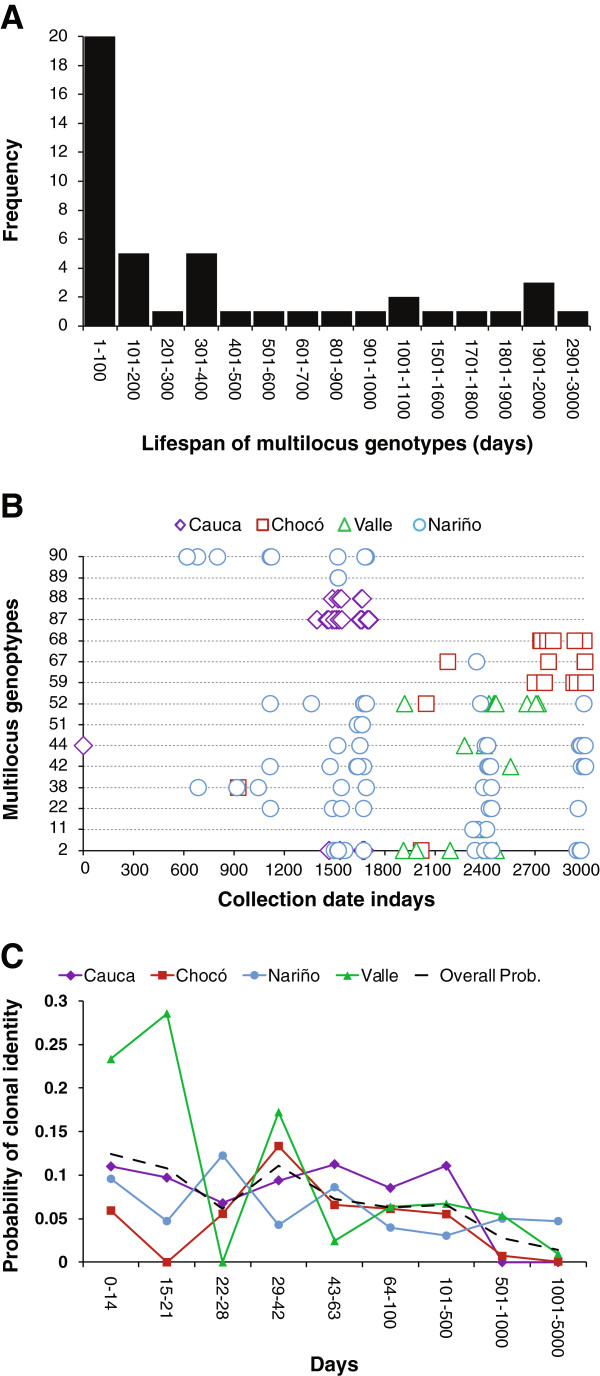
**Analyses of clonality and persistence in multilocus genotypes from Colombian Pacific region. A)** Distribution of persistence times in the whole dataset analyzed (325 samples in 136 MLGs). **B)** Fifteen *P. falciparum* MLGs from Colombian Pacific coast revealed clones from different locations at same MLG and clonal persistence > eight years. **C)** Probability of sampling parasites from the same MLG in each of the four provinces in the Colombian Pacific region and overall probability. The first five intervals of days represent days of follow up during therapeutic efficacy studies with antimalarial drugs.

The sampling period (03/15/1999 to 07/09/2007) covers >3000 days. Using the whole dataset, we observed 45 MLGs that persisted for more than one day (Figure 5A). We examined the clonal persistence in time and space of the 15 most common MLGs (range of 5 – 28 clones per MLG) (Figure 5B). Eight MLGs (n = 67 parasites) were restricted to a single province, while seven MLGs (n = 92 parasites) were recovered from more than one province. A surprisingly long persistence was found with a median of 537.5 (range 1 – 2997) days. Thus, our data suggest that parasite clones persist through the Pacific region for up to eight years (Figures 5A and 5B). This is equivalent to 48 parasite generations assuming six generations per year [31].

In the four locations, the probability of sampling the same MLG decayed slowly and remained between 0.03 and 0.11 at time intervals <500 days (Figure 5C). In Valle, probabilities of sampling identical MLGs were closed to 30% for patients sampled within the first 21 days.

### Non panmitic population in *P. falciparum* samples from Colombian Pacific region

From the STRUCTURE input file, the mean Var [LnP(D)] showed an unimodal distribution reaching a plateau at *K* = 4 (Figure 4B) and a relatively constant α value after *K* = 4, stabilizing at <0.1, suggesting four different subpopulations (Figure 4C). This concurs with Evanno’s methodology [27] using the *K* vs. *ΔK* (Additional file 3). These findings reject panmixia in the Colombian parasites analyzed. The four subpopulations exhibit significant differentiation with *F*_*ST*_ values ranging from 0.084 - 0.279 (Additional file 4). The subpopulation Col–1 is represented by 82 isolates in 46 MLGs, and were highly prevalent in the North Pacific region (Chocó province) with a frequency >80% decreasing dramatically to the South; in contrast, subpopulation Col–4 with 87 isolates in 21 MLGs was highly prevalent in Cauca (>70%) and its frequency was lower to the Pacific North. Subpopulations Col–2 with 86 isolates in 55 MLGs and Col-3 (70 isolates in 14 MLGs) showed a more heterogeneous distribution, comparable between Valle and Nariño, although Col-2 and Col-3 had the higher frequency (>40%), respectively (Figure 4D); this suggests a high chance of parasite admixture in these provinces. As expected, the lowest differentiation between the provinces was found between Valle - Nariño (*F*_*ST*_ = 0.023) while the highest was for Cauca– Chocó (*F*_*ST*_ = 0.117) (Additional file 4). The UPGMA tree based on allele sharing distances showed strong support for the four clusters (Figure 4A).

### Extent of linkage disequilibrium

**Figure 6:**
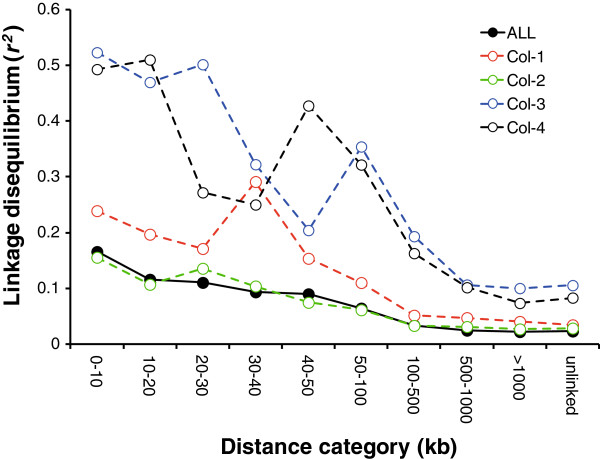
**Linkage disequilibrium analyses in the four subpopulations and the complete dataset.** Linkage disequilibrium measure (r^2^) vs. physical distances between SNPs using the four subpopulations determined by STRUCTURE software (Col-1 to Col-4) and all subpopulations as a whole.

We computed LD for the entire population and for the four subpopulations of parasites identified by STRUCTURE (Figure 6). In the entire population of unique haplotypes (n = 136), the mean r^2^ was 0.16 for markers spaced <10 kb apart and decayed to background levels within ~240 kb. We observed striking differences in the decay of LD values in the four subpopulations (Figure 6), decaying slower in subpopulation Col-3 and Col-4. Within subpopulations Col-3 (n = 14) and Col-4 (n = 21) mean r^2^ was ~0.5 at inter-marker distances <20 kb, complete pairwise LD (r^2^ = 1) was observed for many marker pairs at distances up to ~ 1200 kb, the mean r^2^ decayed to background levels in ~ 500 kb. In addition, Col-3 and Col-4 showed high background levels of LD (r^2^ = 0.11 and 0.08 respectively) between markers on different chromosomes.

The subpopulation Col-2 (n = 55) exhibited the most rapid decay in LD, and patterns of decay for this subpopulation mirrored those observed in the whole population (Figure 6).

## Discussion

Successful genotyping of 307 SNPs in 400 *P. falciparum* infections from four provinces in the Colombian Pacific region over a ten year period allows detailed description of parasite population genetics in this region. These data reveal (a) parasite MLGs persisting for up to eight years (median of 538 days), (b) stratification of parasites into four subpopulations that occur sympatrically within sampling locations, (c) LD decays by half in <10 kb, but varies between subpopulations. We discuss the advantages of the SNP genotyping method used, and the implications of our findings for design of association studies and evolution of drug resistance in low endemic malaria areas.

### Effective genotyping using dried blood spots

In the present study, we were able to rapidly score multiple markers from finger prick blood spots with high reproducibility. The SNP markers selected provide a set of 250 informative SNPs for further genetic studies at the local/regional level. The major advantage of SNPs over microsatellites is that they are more abundant, mutationally stable, located in genes and “portable”; in other words, they are easily scored and comparable between studies.

### Clonality and persistence of MLGs in time and space

Genetic variability studies show a direct relationship between degree of parasite endemicity and genetic variation [32,33]. In high endemic malaria areas, such as sub Saharan Africa, multiclonal *P. falciparum* infections, high genetic diversity, and low LD are common. In contrast, in low endemic areas, such as South American countries, malaria patients are expected to have infections caused by a single clone with limited genetic diversity and more extensive LD [8]. We found that 19% of *P. falciparum* infections were multiclonal in samples collected from the Colombian Pacific region. This is consistent with previous studies in low endemic malaria areas and contrasts with studies in Africa where the percentage of multiclonal infections can reach up to 90% [34]. This correlation between multiclonal infections and transmission intensity was confirmed here within low endemic areas (Figure 3), suggesting that this metric provides an indirect measure of transmission intensity [35,36].

A decade ago, Anderson et al., using 12 microsatellites, stated that *P. falciparum* from South America had the lower level of genetic diversity worldwide, with 30 Colombian samples (collected in Antioquia province), showing the lowest diversity [8]. Another study using 56 samples from Chocó and five polymorphic microsatellites, also suggested low diversity [5].

The genotypic richness (R) [22] was 0.42 for all the monoclonal samples included in this study, the lowest reported in comparison with other studies from similar malaria eco-epidemiological features such as Venezuela, Peru, Brazil, Cambodia and Thailand with R values of 0.60 – 0.98 [9,11,17,18,37-39]; however this measure is strongly influenced by sampling intensity and hence comparisons between countries may be biased [35]. Our study confirmed low genotype richness in *P. falciparum* from Colombia, with a third of the MLGs infecting ≥2 patients (Figure 4A) and long persistence (Figure 5A) in cities separated up to more than 500 km (Figure 1). Our results contrast with studies from neighboring countries including Venezuela, Brazil and Peru, where genotype richness is markedly higher and the number of polyclonal infections has increased (more than double) from 2003 to 2007 [9,38,40].

### Implications for drug efficacy studies

PCR genotyping of parasite infections before and after treatment is widely used to differentiate between reinfection and recrudescence and to adjust measures of treatment failure rates in antimalarial drug efficacy studies [41,42]. However when parasite populations are highly inbred, there is high probability of patients being reinfected with the same parasite genotype [43]. To evaluate this probability in Colombian samples, we examined the probability of sampling identical genotypes. In Valle province, this probability was the highest, during the interval of 15–21 days (~30%), followed by Chocó (29–42 days) and Nariño (22–28 days) with a probabilities close to 15% and Cauca (43–63 days) ~10%. The overall population mean probabilities were between 3 - 11% for up to 500 days (Figure 5C). These results suggest that PCR evaluation should be used with care in Colombia, because there is a strong probability of misclassifying some new parasite infections as recrudescences, thereby overestimating treatment failure rates (Figure 5C).

Colombia implemented Artemisinin Combination The-rapies (ACTs) at the end of 2006. Three drug efficacy studies were performed with these compounds in Antioquia and Chocó provinces, showing 99 to 100% of efficacy[44-47]. One subject from the rural area of Tadó (in Chocó) presented parasitemia and fever at day 28 post treatment with Coartem®; further genetic analyses of the *msp1* gene suggested a recrudescence [47]. However, it is unclear whether this was a true treatment failure, or a case of reinfection with the same genotype. The use of even more polymorphic markers is not going to overcome the limitations of PCR in this scenario. Three alternative approaches could be used to aid interpretation of such studies: i) the use of statistical approaches designed to account for this bias, ii) definition of primary efficacy in terms of parasite clearance rates and iii) the use of “malaria-free locations” for malaria patients during post treatment surveillance [43,48].

### Strong genetic structure in the Colombian Pacific

Both allele sharing methods and Bayesian clustering define four subpopulations and mixed ancestry in the area of study (Figure 4), suggesting that our population structure results are robust. This is in line with previous studies performed in Brazil and Peru, where three to five subpopulations were revealed [9,10,39]. The presence of para-site subpopulations in the Colombian Pacific coast may be partially explained by the bottleneck in *Plasmodium* populations, approximately 9,000 cases in 1960, produced by the implementation of malaria control strategies [49] and subsequent focal reemergence of parasites with different genetic backgrounds.

The coexistence of different sub-populations within locations is consistent with limited genetic exchange, together with extensive migration among locations.*Plasmodium falciparum* genetic interchange in Colombia was suggested recently for parasites through the Andean mountains and North and South of the Pacific region [50]. Identification of identical MLGs in different sampling locations also demonstrates migration of parasite genotypes without breakdown due to recombination. An epidemiological study performed in Quibdó with 670 *P. falciparum* infected patients, revealed that 66% of the cases are from the urban and rural area of the city, while the 33 and 1% are from neighboring municipalities and provinces, respectively [5].

Local adaptation to different vectors may play a role in the parasite population structure. For example, coadaptation between vector and parasite has been suggested for mosquitoes and *Plasmodium vivax* in Mexico [51], where subpopulations of parasites differentially infected *Anopheles albimanus* and *An. pseudopuntipennis*. Three primary and three secondary vectors are found in the Pacific region (Table 1), and at least five different ecological sub-regions had been identified (http://www.eoearth.org/). *Anopheles* populations in Colombia vary locally in their vectorial competence, breeding habitats, and feeding preferences [13]. One possible explanation is that parasite population structure in the Pacific region is also shaped by geographic restriction of compatible vectors. For example, parasites from Col-1 subpopulation may be adapted to *An. darlingi* in Chocó, since this vector had not been registered in the other provinces of the Colombian Pacific region [13,14]. Further experimental investigation is necessary to test this hypothesis. The presence of *An. darlingi* in Chocó, could explain the higher number of multiclonal infections (25%), as this species is considered the most effective malaria vector in Latin America [13,14].

The model of metapopulation structure in *P. falciparum* suggests the potential for spreading of drug resistance alleles [52,53]. Parasites from Colombia may follow this model as they show no panmixia and inbreeding. This fact highlights the need to closely monitor the efficacy of ACTs in Colombia and neighboring countries, since emergence of drug resistance to different antimalarials occurred and disseminated rapidly in this region [2]. Artemisinin resistance has been confirmed in Southeast Asia [54], an area with similar low transmission conditions as South America. Finally, the presence of *P. falciparum* subpopulations in the Colombian Pacific region could explain the different patterns of drug susceptibility (*in vivo* and *in vitro*), as the magnitude of resistance to amodiaquine, sulphadoxine-pyrimethamine, and mefloquine varies between the South and North of this region [50,55].

### Implications for association studies

Association studies require considerable investment of time and resources. Therefore, it is critical to first demonstrate that the traits of interest have a genetic basis and to quantify the heritability in order to calculate appropriate sample sizes [17,18]. Colombian *P. falciparum* populations are well suited to study the heritability of a trait of interest as the estimation of this parameter is achievable when identical clones in populations have been identified.

Colombian *P. falciparum* samples represent a challenge for association studies owing to strong population structure, and presence of many identical or closely related genotypes. In this study there were 136 unique genotypes among 400 parasites sampled. Hence, only a third of parasites sampled would be informative for association analyses. Both population stratification and cryptic relatedness can generate spurious associations [56]. On the other hand, the low numbers of multiple clone infections simplifies detection of genotype/phenotype associations. Both sampling and statistical approaches can minimize bias in this situation. A two phase sampling strategy provides one possible approach to minimize cost and effort, while maximizing study power. In phase one, preliminary genotyping of the parasite population using 96–384 SNPs can rapidly identify identical clones and multiple clone infections. In phase two, a single representative of each clone can be genotyped using higher densities of SNPs using Illumina sequencing [57] or microarray based approaches [58] and characterized for the trait of interest. From the statistical standpoint, powerful mixed model approaches developed by plant geneticists allow for effective control of both stratification and cryptic relatedness [59]. This methodology was recently used to establish the role of the *PF10_0355* membrane protein in low susceptibility to arylaminoalcohols antimalarials [29]. These approaches must be considered in areas of low transmission for *P. falciparum* association studies.

Offspring with limited to zero recombination are expected to occur in South America [60]. Subpopulations Col-3 and Col-4 exhibit the lower genotypic richness (R) with R ~ 0.21, leading to higher LD in comparison with the other populations. Subpopulations Col-1 and Col-2 both with R of ~ 0.59, are likely older and have more likelihood of experiencing recombination. In our samples we estimated persistence up to 48 generations, which reflects transmission over many generations of segments of ancestral haplotypes comprising linked markers.

Despite evidence for high levels of inbreeding, the extent of LD observed was lower than observed in other studies of South American populations. We observed mean r^2^ value of 0.16 between markers spaced <10 kb apart in the whole data set (Figure 6), while Neafsey et al. 2008 reported mean r^2^ of 0.7 for markers spaced <10 kb apart for samples from Brazil [61]. Strong artifactual LD can be generated by combining subpopulations with differing allele frequencies in a single population sample. We therefore expected that LD would be elevated in the total population relative to the individual subpopulations. In fact, we observed the opposite (Figure 6).

The extent of LD varies among the four subpopulations. Mean r^2^ for intermarker distances <10 kb range from 0.15 for Col-2 to 0.52 in Col-3 (Figure 6). Linkage disequilibrium in Col-3 and Col-4 also decays to background levels (r^2^ between markers on different chromosomes) at ~500 kb, more gradually than for Col-1 and Col-2 (Figure 6). Mixing with other parasite populations could also explain the rapid decay in LD in the Col-2 subpopulation. The Col-2 subpopulation is dominant in Buenaventura (Valle State) the most important Colombian port in the Pacific Ocean. The extensive movement of people through this port may increase the chance of parasite admixture.

Several factors may contribute to the elevated LD observed in Col-3 and Col-4. First, these subpopulations are small (n = 14 and 21 for Col-3 and col-4 respectively) so the extended LD may be an artifact of low sample size. Three observations are consistent with this. First, background levels of LD (between unlinked markers on different chromosomes) are much higher in Col-3 and Col-4 than in Col-1 and Col-2. Second, random resampling of 14 haplotypes from the total sample of unique genotypes (n = 136) increased values of *r*^*2*^ by an average of 0.06 in each distance category*.* Third, relatedness or recent admixture may contribute to elevated LD in Col-3 and Col-4. These subpopulations show lower expected heterozygosity (H = 0.25 for Col-3 and H = 0.21 for Col-4) compared with Col-1 (H = 0.27) and Col-2 (H = 0.34), suggesting that they may contain closely related parasites. Finally, genotypic richness is lower in Col-3 and Col-4 (R = 0.19 and 0.25 respectively) compared with Col-1 and Col-2 (R = 0.57 and 0.61 respectively). We suggest that observations of differences in LD decay within parasite subpopulations should be viewed with caution unless artifactual effects of sample size and relatedness can be clearly rejected.

A feature of the LD information is important for association mapping. Only 2.4% of markers situated within 10 kb show r^2^ ≥ 0.8 in the whole dataset. Hence, even in this low transmission region, genome sequencing or efficient genotyping of tagging SNPs will be needed to avoid false negative associations. On the other hand, the rapid decay in LD should enable localization of causative SNPs to genome regions containing 1–5 genes.

## Conclusion

Our study shows the impact of low genotypic richness, persistence of MLGs and population structure of *P. falciparum* on the establishment, distribution and propagation of MLGs in low endemic malaria areas. These features have important implications for the design of ACT clinical efficacy trials and genotype/phenotype association studies. SNP surveys such as these, using moderate numbers of markers, will be critical for maximizing the power, and minimizing bias, in association studies in similar endemic areas in South America using genome sequencing [57] or high resolution microarray methods [58].

## Abbreviations

SNP: Single nucleotide polymorphism; LD: Linkage disequilibrium; MLG: Multilocus genotype; *F*_*st*_: Fixation index; CQ: Chloroquine; Msp: Merozoite surface protein; CIDEIM: International Center for Medical Research and Training; MR4: Research and Reference Reagent Resource Center; WGA: Whole genome amplification; UPGMA: Unweighted pair group method with arithmetic mean; MCMC: Markov chain Montecarlo; MAF: Minor allele frequency; ACT: Arthemisinin combinatory therapy.

## Competing interests

The authors declare that they have no competing interests.

## Authors’ contributions

DFE: Participated in the design of study. Performed genotyping experiments, data analyses and writing of the manuscript. SN: Participated in the supervision of the study, genotyping experiments, data analyses and critically review the manuscript. LO: Participated in the design of the study and writing of the manuscript. SM: participated in genotyping experiments, data analyses and critically review the manuscript. CM: Participated in data analyses and critically review the manuscript. TJCA: Participated in the design of study and implementation of the genotyping assays. Participate in the supervision of the study, data analyses and writing of the manuscript. All authors read and approved the final manuscript.

## Supplementary Material

Additional file 1 **Single nucleotide polymorphisms (SNPs) used for genotyping Colombian P. falciparum samples from the Pacific region.** Detailed information of the 384 coding SNPs used for the GoldenGate® genotyping assay including SNP location, synonymous or non-synonymous SNP status, gene information and summary of the performance of each SNP during the genotyping assay are shown. Click here for file

Additional file 2 **Validation of *P. falciparum *genotyped SNPs using reference strains (controls) performing comparison between them and against PlasmoDB SNPs data.** Bead Studio package output comparisons between technical replicates for reference strains (Dd2, HB3, 7G8 and Santa Lucia) and comparisons between genotyped reference samples (n = 9) and SNP alleles from PlasmoDB. Alleles expected for each SNP are shown in the SNP/OPA column. Alleles expected for the *P. falciparum* reference genome (3D7 strain) and the major and minor allele are also shown. Click here for file

Additional file 3 ***K* value determination using the Evanno’s approach. Δ*K* vs. *K* probability plot following Evanno’s approach.** The analysis suggest the best *K* value at *K* = 4, suggesting four subpopulations of *P. falciparum* parasites from the Colombian Pacific region. Click here for file

Additional file 4 **Pairwise fixation indexes in the Colombian Pacific coast *P. falciparum *samples.** A) *F*_*st*_ values between subpopulations identified by STRUCTURE software and B) *F*_*st*_ values between provinces. The F_st_ values were computed using the GENALEX software [62]. Click here for file
