# Supplementary material for: Long term persistence of clonal malaria parasite Plasmodium falciparum lineages in the Colombian Pacific region
Source: BMC Genet. 2013 Jan 7;14:2. doi: 10.1186/1471-2156-14-2 (PMC3563461; doi:10.1186/1471-2156-14-2)
Supplement: Additional file 4 — Pairwise fixation indexes in the Colombian Pacific coast P. falciparum samples. A) Fst values between subpopulations identified by STRUCTURE software and B) Fst values between provinces. The Fst values were computed using the GENALEX software [62]. [file 1471-2156-14-2-S4.docx]

**A**

| COL-1 | COL-2 | COL-3 | COL-4 |  |
| --- | --- | --- | --- | --- |
| 0 |  |  |  | COL-1 |
| 0.084 | 0 |  |  | COL-2 |
| 0.279 | 0.209 | 0 |  | COL-3 |
| 0.172 | 0.105 | 0.277 | 0 | COL-4 |
|  |  |  |  |  |

**B**

| Cauca | Chocó | Nariño | Valle |  |
| --- | --- | --- | --- | --- |
| 0 |  |  |  | Cauca |
| 0.117 | 0 |  |  | Chocó |
| 0.096 | 0.106 | 0 |  | Nariño |
| 0.073 | 0.076 | 0.023 | 0 | Valle |
|  |  |  |  |  |
